# Mental Well-being of Medical Students in the Visegrad Four Countries: A Cross-Sectional Exploratory Study

**DOI:** 10.1192/j.eurpsy.2025.488

**Published:** 2025-08-26

**Authors:** B. Gács, A. Matuz, Z. Varga

**Affiliations:** 1 Department of Behavioural Sciences, University of Pécs, Medical School, Pécs, Hungary

## Abstract

**Introduction:**

The mental health of medical students is a critical concern, as their well-being directly influences academic performance and the overall success of educational institutions. The high academic demands, heavy workload, and emotional stress encountered by medical students can lead to significant mental strain, potentially resulting in mental disorders. Understanding these factors is essential for developing effective support mechanisms.

**Objectives:**

This study aimed to investigate the mental well-being of medical students across the Visegrad Four countries (Hungary, Czech Republic, Poland, Slovakia) by identifying key predictors of well-being and categorizing students into well-being clusters based on psychological and physical health indicators.

**Methods:**

A cross-sectional exploratory study was conducted using an anonymous, English-language online questionnaire. The survey gathered general demographic data, health-related information, and academic attitudes. Mental well-being was assessed using the Warwick-Edinburgh Mental Well-being Scale (WEMWBS), coping strategies were evaluated with the Brief COPE inventory, and somatic symptoms were measured using the Patient Health Questionnaire-15 (PHQ-15). Regression analysis was performed to identify predictors of mental well-being, and a two-step cluster analysis was employed to classify students into distinct well-being groups.

**Results:**

A total of 1,703 medical students (467 males) participated in the study. Regression analysis identified adaptive problem-focused and emotion-focused coping, social support, satisfaction with the university experience, healthy eating habits, and a sense of control over personal health as positive predictors of mental well-being. In contrast, maladaptive coping strategies (avoidant and passive) and frequent somatic symptoms were negative predictors. The cluster analysis revealed three distinct groups: (1) a stable group with high well-being and satisfaction, low somatic symptom frequency, and low incidence of mental disorders; (2) a risk group with moderate well-being, low satisfaction, higher somatic symptom frequency, and increased incidence of mental disorders; and (3) a problematic group characterized by low well-being, low satisfaction, high somatic symptom frequency, and frequent mental disorders.

**Conclusions:**

The findings suggest that enhancing adaptive coping strategies, the sense of control, and perceived social support may significantly improve mental well-being of medical students. Furthermore, identifying risk and problematic groups can support the development of targeted interventions. These insights not only contribute to a better understanding of medical students’ mental well-being but also offer practical implications for designing preventive and supportive programs to address mental disorders.

**Disclosure of Interest:**

None Declared

